# Supplementary material for: Resolving the full spectrum of human genome variation using Linked-Reads
Source: Genome Res. 2019 Apr;29(4):635–45. doi: 10.1101/gr.234443.118 (PMC6442396; doi:10.1101/gr.234443.118)
Supplement: Supplemental Material [file supp_gr.234443.118_Supplemental_longranger-2.2.zip › longranger-2.2.tar.bz2!/lr2.2-public/lib/stan/cmdstan/stan/doc/api/html/arg__optimize__algo_8hpp_source.html]

Stan: src/stan/gm/arguments/arg\_optimize\_algo.hpp Source File


|  |  |
| --- | --- |
|  | Stan  2.5.0  probability, sampling & optimization |


- Main Page
- Related Pages
- Namespaces
- Classes
- Files

- File List
- File Members

All Classes Namespaces Files Functions Variables Typedefs Enumerations Enumerator Friends Macros Pages

- src
- stan
- gm
- arguments

arg\_optimize\_algo.hpp

Go to the documentation of this file.

1 #ifndef STAN\_\_GM\_\_ARGUMENTS\_\_OPTIMIZE\_\_ALGO\_\_HPP

2 #define STAN\_\_GM\_\_ARGUMENTS\_\_OPTIMIZE\_\_ALGO\_\_HPP

3

4 #include <stan/gm/arguments/list\_argument.hpp>

5

6 #include <stan/gm/arguments/arg\_bfgs.hpp>

7 #include <stan/gm/arguments/arg\_lbfgs.hpp>

8 #include <stan/gm/arguments/arg\_newton.hpp>

9

10 namespace stan {

11

12  namespace gm {

13

14  class arg\_optimize\_algo: public list\_argument {

15

16  public:

17

18  arg\_optimize\_algo() {

19

20  \_name = "algorithm";

21  \_description = "Optimization algorithm";

22

23  \_values.push\_back(new arg\_bfgs());

24  \_values.push\_back(new arg\_lbfgs());

25  \_values.push\_back(new arg\_newton());

26

27  \_default\_cursor = 1;

28  \_cursor = \_default\_cursor;

29

30  }

31

32  };

33

34  } // gm

35

36 } // stan

37

38 #endif

39

stan::gm::arg\_optimize\_algo

**Definition:** arg\_optimize\_algo.hpp:14

stan::gm::list\_argument::\_default\_cursor

int \_default\_cursor

**Definition:** list\_argument.hpp:183

stan::gm::arg\_lbfgs

**Definition:** arg\_lbfgs.hpp:12

list\_argument.hpp

stan::gm::list\_argument::\_values

std::vector< argument \* > \_values

**Definition:** list\_argument.hpp:185

stan::gm::arg\_optimize\_algo::arg\_optimize\_algo

arg\_optimize\_algo()

**Definition:** arg\_optimize\_algo.hpp:18

stan::gm::list\_argument

**Definition:** list\_argument.hpp:12

stan::gm::arg\_newton

**Definition:** arg\_newton.hpp:10

stan::gm::argument::\_name

std::string \_name

**Definition:** argument.hpp:76

stan::gm::arg\_bfgs

**Definition:** arg\_bfgs.hpp:13

stan::gm::list\_argument::\_cursor

int \_cursor

**Definition:** list\_argument.hpp:182

arg\_lbfgs.hpp

arg\_bfgs.hpp

stan::gm::argument::\_description

std::string \_description

**Definition:** argument.hpp:77

arg\_newton.hpp


---

[ Stan Home Page ]

*© 2011–2014,
Stan Development Team.*
